# Prognosis and diagnosis of prostate cancer based on hypergraph regularization sparse least partial squares regression algorithm

**DOI:** 10.18632/aging.205889

**Published:** 2024-05-31

**Authors:** Ruo-Hui Huang, Zi-Lu Ge, Gang Xu, Qing-Ming Zeng, Bo Jiang, Guan-Cheng Xiao, Wei Xia, Yu-Ting Wu, Yun-Feng Liao

**Affiliations:** 1 Department of Urology, First Affiliated Hospital of Gannan Medical University, Ganzhou, Jiangxi, China; 2 First Clinical Medical College, Gannan Medical University, Ganzhou, Jiangxi, China

**Keywords:** prostate cancer, DNA methylation, prognosis, diagnosis, biomarkers

## Abstract

Background: Prostate cancer (PCa) is a malignant tumor of the male reproductive system, and its incidence has increased significantly in recent years. This study aimed to further identify candidate biomarkers with prognostic and diagnostic significance by integrating gene expression and DNA methylation data from PCa patients through association analysis.

Material and methods: To this end, this paper proposes a sparse partial least squares regression algorithm based on hypergraph regularization (HR-SPLS) by integrating and clustering two kinds of data. Next, module 2, with the most significant weight, was selected for further analysis according to the weight of each module related to DNA methylation and mRNAs. Based on the DNA methylation sites in module 2, this paper uses multiple machine learning methods to construct a PCa diagnosis-related model of 10-DNA methylation sites.

Results: The results of Receiver Operating Characteristic (ROC) analysis showed that the DNA methylation-related diagnostic model we constructed could diagnose PCa patients with high accuracy. Subsequently, based on the mRNAs in module 2, we constructed a prognostic model for 7-mRNAs (MYH11, ACTG2, DDR2, CDC42EP3, MARCKSL1, LMOD1, and MYLK) using multivariate Cox regression analysis. The prognostic model could predict the disease free survival of PCa patients with moderate to high accuracy (area under the curve (AUC) =0.761). In addition, Gene Set EnrichmentAnalysis (GSEA) and immune analysis indicated that the prognosis of patients in the risk group might be related to immune cell infiltration.

Conclusions: Our findings may provide new methods and insights for identifying disease-related biomarkers by integrating DNA methylation and gene expression data.

## INTRODUCTION

Prostate cancer (PCa) is the most common cancer in older men [1]. Serum prostate-specific antigen screening (PSA) is a common method for early diagnosis of PCa. However, the sensitivity and specificity of the PSA test remain low [2]. Therefore, from the perspective of bioinformatics, it is necessary to design and develop an association analysis method for PCa-related transcriptomic data to identify significant biomarkers related to diagnosis.

Li and colleagues analyzed the metabolic phenomenon in PCa, established the prognostic features based on PCa tyrosine metabolism-related genes, and provided a reference for its treatment and prevention [3]. From a bioinformatics perspective, Wo et al. explained the effects of ferritinopathies on the ferroptosis potential index (FPI), high and low FPI groups, gene mutations, and various cell signaling pathways [4]. The critical role of autophagy in PCa progress and treatment resistance has been preliminarily revealed. Wen and others have chosen six autophagy-related genes to establish characteristics, predict the prognosis of PCA patients, and obtain high accuracy [5]. The abnormal expression of N6-METHYLADENOSINE (M6A) is significantly related to cancer progress and immune cell infiltration. The role of these regulatory factors in PCa is still being determined. Liang and others checked the expression spectrum and methylation level of 21 M6A, built a diagnostic model and found the potential biomarkers of PCa [6]. Coking disease is closely related to the tumor microenvironment (TME) and immune infiltration. Wang and colleagues discussed the relationship between PCa, coking disease, TME, and tumor immunohism [7]. Li et al. proposed a stable feature selection method (StabML-RFE) to screen robust biomarkers. StabML-RFE takes some popular ML-RFE methods and integrates them into an aggregation-like framework. The algorithm integrates best feature subsets by aggregating area under the curve (AUC) values and stability indices. This method can screen and obtain robust biomarkers [8].

The above analysis was performed only on the transcriptome data of PCa. However, DNA methylation data also plays a vital role in PCa. They may carry complementary information to transcriptome data. Wei et al. developed a deep learning approach to identify differentially expressed genes (DEGs) of PCa, enrichment pathway analysis, copy number analysis, and immune cell infiltration analysis [9]. Qiu et al. proposed a JONMF algorithm to integrate Long non-coding RNA and Messenger RNA expression profiles of ovarian cancer samples to identify lncRNA-mRNA co-expression modules. The model adopts orthogonal non-negative matrix decomposition, effectively preventing multicollinearity and producing highly interpretable results [10]. In addition, sparse partial least squares regression (SPLS) is another commonly used association analysis algorithm, which studies the association between data types by maximizing the covariance between their corresponding latent variables [11]. The SPLS algorithm adds the l1 norm of the weight vector to the objective function, which is more conducive to analyzing high-dimensional data. However, this method does not consider the network structure inside the two data. Chen et al. proposed a Sparse Network Regularized Partial Least Squares Regression (SNPLS) algorithm that incorporates Laplacian regularization constraints on the data to predict the relationship between genes and drug responses. To a certain extent, the interpretability of the results is improved [12]. A hypergraph is an extension of a simple graph. In this paper, we add hypergraph regularization to the SPLS algorithm and propose a sparse partial least squares regression (HR-SPLS) algorithm based on hypergraph regularization. Hypergraph regularization can identify the high-order associations within PCa patient genes, and methylation data enable the algorithm to deeply identify genes and methylation sites with potential relationships. The results show that the HR-SPLS algorithm can identify biomarkers closely related to the diagnosis and prognosis of PCa, and provide a reference for the early prevention and diagnosis of PCa.

## MATERIALS AND METHODS

### Data source

We downloaded gene expression data of prostate cancer patients (TCGA-PRAD) from the TGCA database (https://portal.gdc.cancer.gov/), which included 499 prostate cancer tissue samples and 52 normal tissue samples. The methylation data and corresponding clinical information of prostate cancer patients in the TCGA-PRAD cohort were downloaded from the UCSC Xena database (https://xenabrowser.net/datapages/). In addition, we downloaded the GSE116918 dataset from the Gene Expression Omnibus (GEO) database for external validation of the prognostic model. A total of 248 patients with prostate cancer who received radical radiotherapy were included in the GSE116918 dataset. We divided the training set and test set by the ratio of about 7:3 on the samples of The Cancer Genome Atlas (TCGA) dataset. Finally, 428 training set samples and 123 test set samples were obtained.

This paper uses transcriptome and methylation data from the same batch of prostate cancer samples for association analysis. The weight vectors of the two data are obtained through the proposed HR-SPLS algorithm. Then the modules are divided according to the set number of modules. Modules with smaller objective function values were selected for various bioinformatic analyses. Finally, the disease samples of the test set and the independent test set GSE116918 data set were used to verify the prognostic-related genes and the model.

### Partial least squares regression (PLS)

The partial least squares algorithm can simultaneously model multiple independent and dependent variables, especially when multicollinearity exists in both. The objective function is as follows.


$$\underset{g,d}{\text{max}}\text{ cov }(Xg,\;Yd)$$

$$\text{s.t. }g^{T}g=1,d^{T}d=1$$ (1)

Among them, $X\in {\mathbb{R}}^{n\times p}$ represents the expression matrix of the first data. $Y\in {\mathbb{R}}^{n\times q}$ represents the expression matrix of the second data. *n* represents the total number of samples. *p* and *q* represent the first and second data characteristics, respectively. cov (·) represents the difference between the co -party. *g* and *d* represent the two right vectors. Further, this article introduces two potential variables: *u* and *v*. Among them, *u* = *Xg*, *v* = *Yd*. Formula (1) The covariance between the two potential variables *u* and *v* through maximizing the two potential variables *u* and *v*.

### Sparse partial least squares regression (SPLS)

PLS does not meet the needs for high-dimensional biological chip data analysis. Therefore, SPLS is proposed to solve the feature selection of high-dimensional data. SPLS adds model punishment items to the model of the suitable vector and, based on PLS, helps the algorithm selection of more representative and essential features. The target function of the SPLS algorithm is shown below.


$$\underset{g,d}{\text{max }}\text{ cov }(Xg,Yd)-\lambda_{\;1}∥g{∥}_{\;1}-\lambda_{\;2}∥d{∥}_{\;1}$$

$$\text{s.t. }g^{T}g=1,d^{T}d=1$$ (2)

Among them, λ_1_ and λ_2_ control the constraint strength of the *l*1 norm of the weight vectors *g* and *d*, respectively. It can be used to select variables with better biological interpretability.

### Hypergraph learning

The simple graph can represent the pair relationship between the objects. The vertex can be expressed as an object, and the edge represents the relationship between the apex. However, the complex relationship may not be represented in a simple graph, which may cause information loss. Hypergraph can connect to two or more vertices through the hyper edge. As an extension of a simple graph, each side of the hyper edge can be connected to multiple vertices, called a hypergraph. *G* (*V*, *E*, *w*) represents hypergraph. Among them, $V=\{v_{1},v_{2},\ldots ,v_{N}\}\in {\mathbb{R}}^{N}$represents the vertex in the hypergraph. $E=\{e_{1},e_{2},\ldots ,e_{M}\}\in {\mathbb{R}}^{M}$ represents the hyper edge in the hypergraph. $w={\left(w(e_{1}),w(e_{2}),\ldots ,w(e_{M})\right)}^{T}\in {\mathbb{R}}^{M}$ is the weight of E. Next, this paper introduces the associated matrix *H* to characterize the relationship between *V* and *E*. The element at row *i* and column *j* in *H* can be expressed as:

$$H_{ij}=\{\begin{array}{ll} 1, & \text{ if }v_{i}\in e\\ 0, & \text{ if }v_{i}\notin e \end{array}$$ (3)

**Figure 1:**
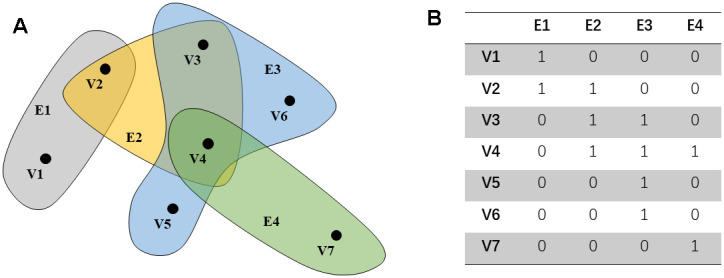
**An example of a hypergraph.** The points in (**A**) represent the distribution of characteristics in the space. Each hyper edge is composed of multiple interconnected data points. (**B**) shows the connection between the super edge and the vertex.

Further, define the degree matrix **D***_v_* and **D***_e_* of the edge and vertex and the diagonal matrix **W**, as shown below. In addition, this article gives a simple hypergraph example (Figure 1A, 1B).

$$D_{v}=\left(\begin{array}{ccc} \sum_{e_{j}\in ℰ}w\left(e_{j}\right)H_{1j} & \cdots & 0\\ \vdots & \ddots & \vdots \\ 0 & \cdots & \sum_{e_{j}\in ℰ}w\left(e_{j}\right)H_{Nj} \end{array}\right)$$ (4)

$$D_{e}=\left(\begin{array}{ccc} \sum_{v_{i}\in V}H_{i1} & \cdots & 0\\ \vdots & \ddots & \vdots \\ 0 & \cdots & \sum_{v_{i}\in V}H_{iM} \end{array}\right)$$ (5)

$$W=\left(\begin{array}{ccc} w\left(e_{1}\right) & \cdots & 0\\ \vdots & \ddots & \vdots \\ 0 & \cdots & w\left(e_{\text{M}}\right) \end{array}\right)$$ (6)

Then this paper defines the similarity matrix **S** of the hypergraph *G* and the Laplacian matrix *L_H_* of the hypergraph.

$$S=HWD_{e}^{-1}H^{T}\in {\mathbb{R}}^{N\times N}$$ (7)

Similar to the definition of the symmetrical Laplace matrix of the simple graph, the symmetry of the hypergraph is defined below.

$$L=I-D_{v}^{\;-\frac{1}{2}}SD_{v}^{\;-\frac{1}{2}}$$ (8)

### Sparse partial least squares regression algorithm based on hypergraph regularization (HR-SPLS)

Hypergraphs can characterize a high-level relationship between complex objects. In this paper, we innovatively added the hypergraph to the SPLS algorithm as the priority information and proposed the HR-SPLS algorithm. Before defining the target function of this paper, first of all, the hypergraph definition of this article is given.

$$\Omega (g)=g^{T}L_{H1}g$$ (9)

$$\Omega (d)=d^{T}L_{H2}d$$ (10)

Among them, *L_H_*_1_ and *L_H_*_2_ are hypergraph Laplace matrix.

$$L_{H1}=I-D_{v1}^{\;-\frac{1}{2}}S_{1}D_{v1}^{\;-\frac{1}{2}}$$ (11)

$$L_{H2}=I-D_{v2}^{\;-\frac{1}{2}}S_{2}D_{v2}^{\;-\frac{1}{2}}$$ (12)

**D***_v_*_1_ and **D***_v_*_2_ represent the degree matrix of *X* and *Y*, respectively, and ***S*_1_** and ***S*_2_** represent the similarity matrix of *X* and *Y*, respectively. We can get the target function of the HR-SPLS algorithm.


$$\begin{array}{c} \underset{g,d}{\text{max }}\text{ cov }(Xg,\;Yd)-\beta_{1}\Omega (g)-\beta_{2}\Omega (d)\\ -\lambda_{1}∥g{∥}_{1}-\lambda_{2}∥d{∥}_{1} \end{array}$$

$$\text{s.t. }g^{T}g=1,d^{T}d=1$$ (13)

Among them, *β*_1_ and β_2_ control the strength of the hypergraph regularization respectively. λ_1_ and λ_2_ control the sparseness of the two weight vectors, respectively. Further, we can rewrite it:

$$\begin{array}{c} \underset{g,d}{min}-\frac{1}{p}g^{T}X^{T}Yd+\beta_{1}\\ \sum_{1\leq i<j\leq n}s_{1_{ij}}{\left(\frac{g_{i}}{\sqrt{l_{H1_{i}}}}-\frac{g_{j}}{\sqrt{l_{H1_{j}}}}\right)}^{2}\\ +\beta_{2}\sum_{1\leq i<j\leq n}s_{2_{ij}}{\left(\frac{d_{i}}{\sqrt{l_{H2_{i}}}}-\frac{d_{j}}{\sqrt{l_{H2_{j}}}}\right)}^{2}\\ +\lambda_{1}∥g{∥}_{1}+\lambda_{2}∥d{∥}_{1}\\ \text{s.t. }g^{T}g=1,d^{T}d=1. \end{array}$$ (14)

Here, $s_{1_{ij}}$ and $s_{2_{ij}}$ represent the *i* – *th* row and *j* – *th* column of the *X* and *Y* similarity matrices ***S*_1_ and *S*_2_**. $l_{H1_{i}}$ and $l_{H2_{j}}$ represent the *i* – *th* row and *j* – *th* column of $l_{H1_{i}}$ and $l_{H2_{j}}$, respectively. Similar to literature [13], this paper uses the coordinate descent algorithm to find the local maximum of this problem by alternately updating the variables *g* and *d*.

The objective of HR-SPLS is to discover a low-dimensional representation containing the most relevant information from both *X* and *Y*. Specifically, the algorithm achieves integration by identifying latent variables, denoted as g and d, between *X* and *Y*. These latent variables are obtained by projecting *X* and *Y* onto a new coordinate system, aiming to maximize their covariance in this new coordinate space $\left(-\frac{1}{p}g^{T}X^{T}Yd\right)$. This ensures the preservation of the most relevant information between *X* and *Y* in the latent variables. The introduction of regularization terms (*l*_1_ norm and hypergraph regularization) mitigates overfitting caused by an excessive number of features, thereby enhancing the model’s generalization capability.

In addition, this paper uses the solution of PLS as the initial solution of the current algorithm. Specifically, any column of *X* and *Y* is first randomly selected as the initial values of *u* and *v*. The following formula is then used to iteratively select the objective function value for *g*, *d*, *u* and *v*.

$$g:=\frac{X^{T}v}{v^{T}v};g:=\frac{g}{∥g{∥}_{2}};u:=Xg$$ (15)

$$d:=\frac{Y^{T}u}{u^{T}u};d:=\frac{d}{∥d{∥}_{2}};v:=Yd$$ (16)

Next, the weight vectors *g* and *d* are updated alternately. First, fix *d*, and obtain the partial derivative of *g* to obtain the iterative update rule of *g*.

$$g_{j}\leftarrow \frac{\text{sign}\left(z_{g}\right){\left(\left|z_{g}\right|-\lambda_{1}\right)}_{+}}{2\left(\beta_{1}+\delta_{1}\right)}\;j=1,2,\ldots ,n(\delta_{1}>0)$$ (17)

Among them, the $z_{g}=t_{g_{j}}+2\lambda_{1}\sum_{i=1}^{n}\frac{s_{ij}g_{i}}{\sqrt{l_{H1_{i}}l_{H1_{j}}}}$, $t_{g}=\frac{1}{p}\left(X^{T}Yd\right)=\frac{1}{p}\left(X^{T}v\right)$. $t_{g_{j}}$ is the *j* – *th* element of *t_g_*. Finally, the iterative update rule of *d* can be obtained by fixing *g* and taking the partial derivative for *d*.

$$d_{j}\leftarrow \frac{\text{sign}\left(z_{d}\right){\left(\left|z_{d}\right|-\lambda_{2}\right)}_{+}}{2\left(\beta_{2}+\delta_{2}\right)}\;j=1,2,\ldots ,n(\delta_{2}>0)$$ (18)

Among them, $z_{d}=t_{d_{j}}+2\lambda_{2}\sum_{i=1}^{n}\frac{s_{ij}d_{i}}{\sqrt{l_{H2_{i}}l_{H2_{j}}}}$, $t_{d}=\frac{1}{p}\left(Y^{T}Xg\right)=\frac{1}{p}\left(Y^{T}u\right)$ is the *j* – *th* element of *t_d_*.

### Module membership confirmation and correlation analysis

After obtaining the final weight vectors *g* and *d*, this paper uses the Z-score method to confirm whether features of *X* and *Y* are eligible to enter the module. Specifically, *g* and *d* are first normalized using the Z-score method to obtain g^*^ and d^*^. Then, the corresponding features whose g^*^ and d^*^ are more significant than the artificially set threshold *T* are selected for subsequent analysis. In addition, this paper normalizes the *l*_2_ norm of u and v and obtains the normalized u^*^ and v^*^. Then normalize (u^*^+v^*^), and select the sample when (u^*^+v^*^)>*T*. The threshold set in this paper is *T* = 1. After running the algorithm to get the first module, this paper subtracts the module signal from the input data:

$$X:=X-up^{T},\;p=\frac{X^{T}u}{u^{T}u}$$ (19)

$$Y:=Y-vq^{T},\;q=\frac{Y^{T}v}{v^{T}v}$$ (20)

To confirm the correlation of two kinds of data in the same module, this paper defines the Pearson correlation coefficient (PCC) as follows.

$$PCC\left(X,Y\right)=\frac{\text{cov}\left(X,Y\right)}{\sigma_{X}\sigma_{Y}}$$ (21)

Among them, *σ_X_* and *σ_Y_* points represent the standard deviation of *X* and *Y*. This paper calculates the Pearson correlation coefficient *PCC* (*u*, *v*) between *Xg* and *Yd* within each module as a measure of module selection. In addition, this paper also introduces the module error to measure the module’s performance, which is defined as follows.

$$\begin{array}{l} ModuLe\_Error\;=\text{p}/n*\sum_{ij}{\left(X-up^{T}\right)}_{ij}^{2}\\ \;+\text{p}/n*\sum_{ij}{\left(Y-vq^{T}\right)}_{ij}^{2} \end{array}$$ (22)

### Survival analysis

We obtained the overall survival (OS) and disease-free survival (DFS) time of PCa patients from clinical data. Kaplan-Meier (KM) analysis was used to screen out the methylation sites associated with OS in PCa patients. Methylation sites with a p-value less than 0.05 were used as the input for constructing a diagnostic model for PCa patients. Univariate Cox regression analysis was used to screen out candidate features associated with DFS in PCa patients. Genes with p-values less than 0.03 were reserved for further research. Then, we constructed PCa-related prognostic models using multivariate Cox regression analysis and calculated risk scores for PCa patients. risk score=(β_mRNA_1_ * expression level of mRNA_1)+ (β_mRNA_2_ * expression level of mRNA_2)+…+(β_mRNA_n_ * expression level of mRNA_n). Based on the median risk score, we divided PCa patients in the TCGA-PRAD cohort and GSE116918 into two risk subgroups. KM curves were used to compare the differences in DFS of patients in the two risk subgroups. We used Receiver Operating Characteristic (ROC) curves to assess the accuracy of prognostic models for predicting 1-, 3-, and 5-year survival in PCa patients. ROC curves are drawn by the “timeROC” package.

### Gene set enrichment analysis (GSEA)

We downloaded the “c2.all.v7.2.symbols.gmt” gene set from the GSEA database (https://www.gsea-msigdb.org/gsea/msigdb/index.jsp). Based on the “c2.all.v7.2.symbols.gmt” gene set, we performed GSEA analysis on high- and low-risk subgroups to identify pathways enriched between the two risk subgroups. The size of the gene set is set from 10 to 500. Gene sets were considered significant pathways when the absolute value of NES was greater than 1.5, p-value < 0.05, and FDR > 0.25.

### Analysis of immune infiltration among risk subgroups

To further explore the immune microenvironment between the two risk subgroups, we performed a Cell-type Identification By Estimating Relative Subsets Of RNA Transcripts (CIBERSORT) analysis using the “e1071” package. CIBERSORT analysis was used to calculate the composition and infiltration levels of 22 immune cells (T cells, B cells, macrophages, dendritic cells, Natural Killer (NK) cells, monocytes, mast cells, eosinophils, and neutrophils) between the two risk subgroups. The Pearson correlation coefficient was used to calculate the correlation between prognosis-related genes and immune cells.

### Data availability

The gene expression data of prostate cancer patients (TCGA-PRAD) were downloaded from the TGCA database (https://portal.gdc.cancer.gov/). The methylation data and corresponding clinical information of prostate cancer patients in the TCGA-PRAD cohort were downloaded from the UCSC Xena database (https://xenabrowser.net/datapages/). In addition, we downloaded the GSE116918 dataset from the GEO database (https://www.ncbi.nlm.nih.gov/geo/) for external validation of the prognostic model.

## RESULTS

### Data preprocessing

**Figure 2:**
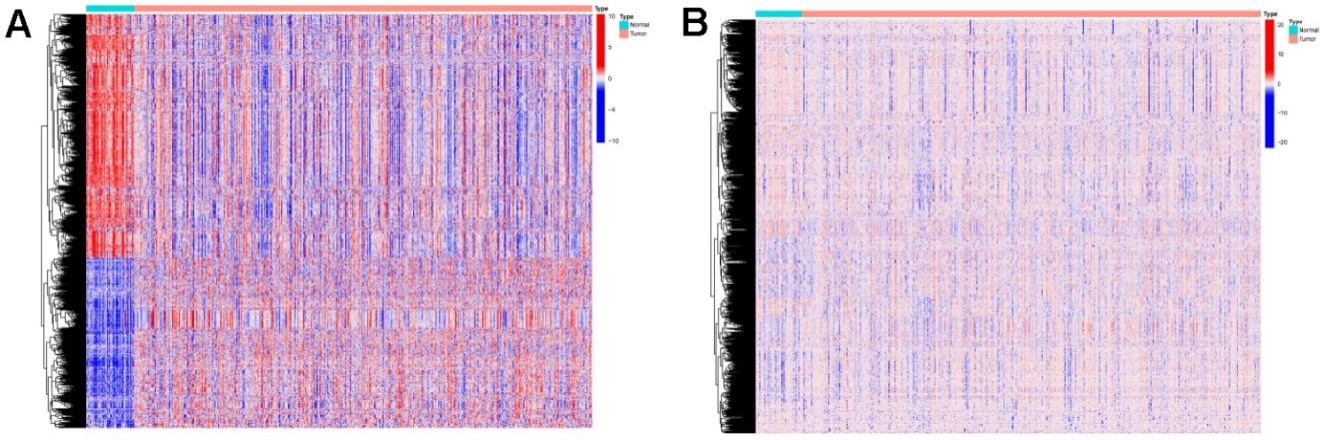
**Expressions of the 1350 mRNAs and 1469 DNA methylation sites.** (**A**) Heatmap (blue: low expression level; red: high expression level) of the 1350 mRNAs between the normal (N, blue) and the tumor tissues (T, red). (**B**) Heatmap (blue: low expression level; red: high expression level) of the 1469 DNA methylation sites between the normal (N, blue) and the tumor tissues (T, red).

The Limma package was used for the differential expression analysis of gene expression data and methylation data on samples from the training set. Genes with absolute logFC values greater than 1 and FDR values less than 0.05 were considered differentially expressed. Methylated sites with FDR values less than 0.05 were considered differentially expressed. We obtained 1350 differentially expressed mRNAs (Figure 2A) and 1469 differentially expressed methylation sites (Figure 2B). Differentially expressed genes and expression profiles of methylation sites were used as input data for the algorithm.

### Selection of hyperparameters

**Figure 3:**
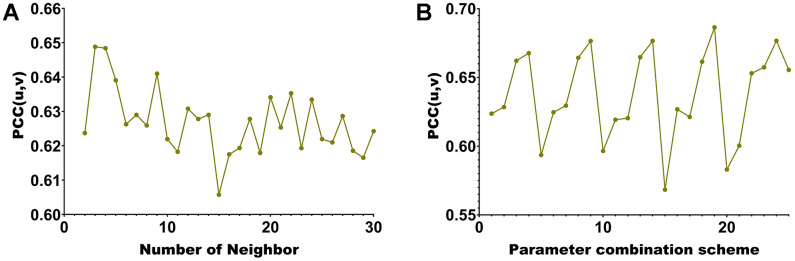
**Hyperparameter selection line chart.** (**A**) corresponding to different number of neighbors of KNN. (**B**) corresponding to different parameter combinations.

This paper selects 20 modules. Each hyperedge’s maximum number of vertices when building the hypergraph is chosen (Figure 3A). Our algorithm incorporates methylated sites and genes with significant associations into the same co-expression module. Consequently, the correlation between two modalities of data within the module can be assessed using Pearson Correlation Coefficient (PCC). A higher PCC indicates a stronger correlation among members within the module, providing additional confirmation of the algorithm’s capabilities in association analysis and feature selection. When the maximum number of vertices is 2, *pcc*(*u*, *v*) is the largest. Further, this paper also uses *pcc*(*u*, *v*) to select the four hyperparameters of λ_1_, λ_2_, *β*_1_ and *β*_2_ from the range of [0.01 0.05 0.1 0.5 1]. We present the PCC (Pearson Correlation Coefficient) of the algorithm for 625 parameter combinations in Figure 3B.

The maximum module correlation can be obtained when the number of neighbors is (Figure 3A). The largest module correlation can be obtained under the 157th set of parameter combinations (Figure 3B). The parameter value corresponding to the 20th group of parameters is λ_1_ = λ_2_ = *β*_1_ = *β*_2_ = 0.5.

### Module description and selection

**Figure 4:**
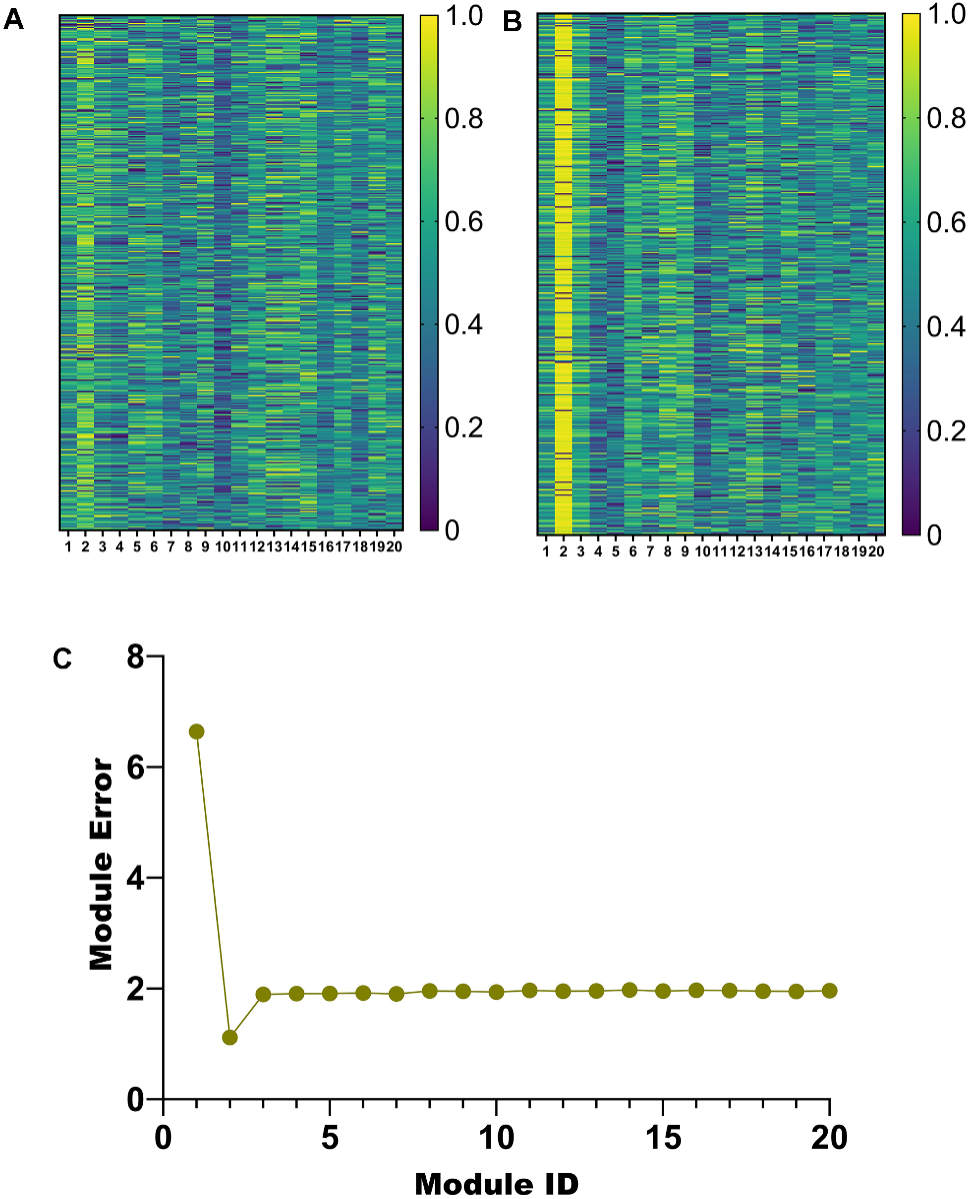
**Module selection.** (**A**) Weight heatmap of u. (**B**) Weight heatmap for v. (**C**) Error line graph for 20 modules.

According to the parameter selection results in Section 3.1, 20 modules are obtained in this paper. To select the salient modules among them, we draw heatmaps of the weights of *u* and *v* corresponding to the 20 modules, respectively (Figure 4A, 4B). In addition, a line graph of module error for the 20 modules was also plotted (Figure 4C).

Among the 20 modules obtained by the proposed algorithm, the weights of *u* and *v* corresponding to module 2 are higher (Figure 4A, 4B). Furthermore, module 2 has a minor error (Figure 4C). Therefore, module 2 will be analyzed in detail later.

### Comparison with other algorithms

**Figure 5:**
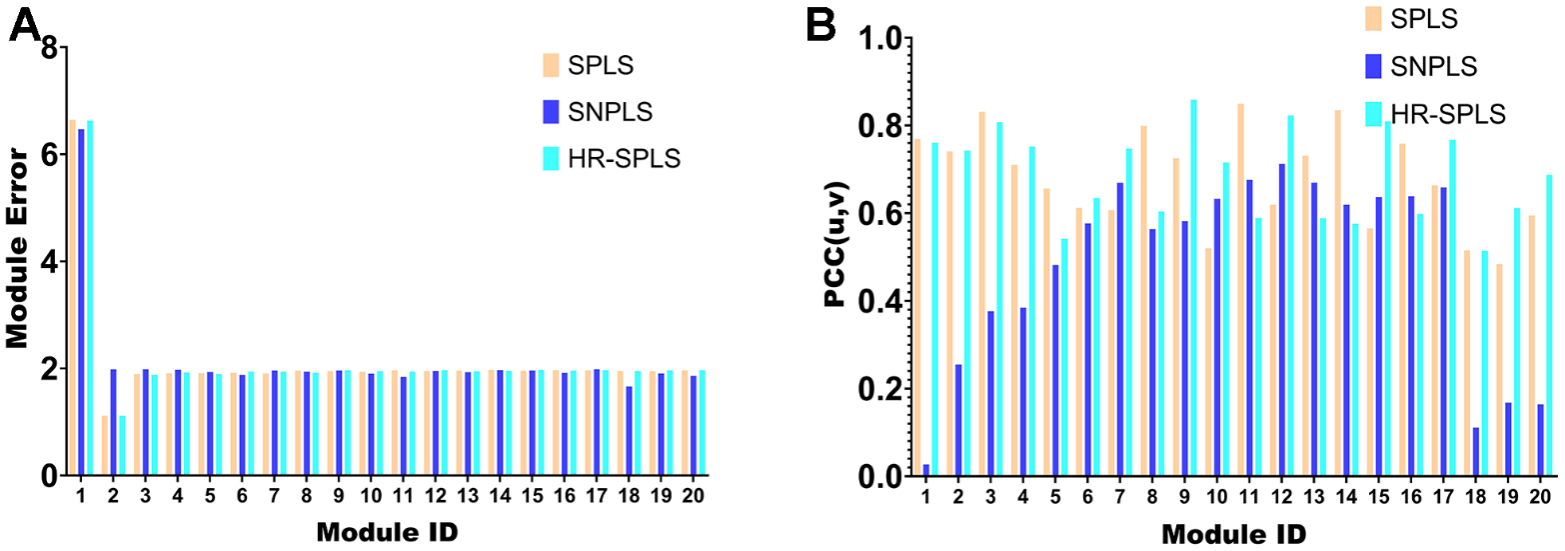
**Algorithmic performance comparison with other algorithms.** (**A**) Module error of 20 modules corresponding to the three algorithms. (**B**) Membership correlations of 20 modules corresponding to the three algorithms.

To confirm the performance of the HR-SPLS algorithm, this paper introduces the SPLS algorithm and SNPLS algorithm to compare the performance of the three algorithms. The module error and membership correlation were used to compare the performance of the 20 modules corresponding to the three algorithms (Figure 5A, 5B).

Further, this paper counts the mean value of the module error and the mean value of the module correlation in the 20 modules of the three algorithms (Table 1). To confirm the importance of sparse constraints for feature selection in high-dimensional omics data, we compared the objective functions of HR-SPLS obtained by adding sparse constraints or not. Under the same number of iterations, the objective function values without and with sparse constraints were 4.3075 and 0.5667, respectively. Therefore, sparse constraints can significantly accelerate the convergence speed of the algorithm and make the performance of the algorithm better.

**Table 1:** The mean value of module error, module correlation and objective function value obtained by three algorithms.

**Algorithm**	**Mean of module errors**	**Mean of module correlations**	**Objective function value**
SPLS	2.1377	0.6794	32.4574
SNPLS	2.1494	0.4803	28.2866
HR-SPLS	2.1381	0.6865	25.7287

The module correlation of HR-SPLS is better than the other two algorithms (Table 1). The mean value of the module error is between the two different algorithms, which further confirms the correlation performance of the algorithm on the two kinds of data.

Additionally, we explored whether elastic net regularization could further enhance the performance of the algorithm proposed in this paper. Specifically, elastic net regularization is a method that combines L1 (Lasso) and L2 (Ridge) regularization. The objective function of applying this regularization to the algorithm in this paper is presented below.

$$\begin{array}{c} \underset{g,\;d}{mim}\;-\frac{1}{p}g^{T}X^{T}Yd+\beta_{1}\\ \sum_{1\leq i<j\leq n}s_{1_{ij}}{\left(\frac{g_{i}}{\sqrt{l_{H1_{i}}}}-\frac{g_{j}}{\sqrt{l_{H1_{j}}}}\right)}^{2}\\ +\beta_{2}\sum_{1\leq i<j\leq n}s_{2_{ij}}{\left(\frac{d_{i}}{\sqrt{l_{H2_{i}}}}-\frac{d_{j}}{\sqrt{l_{H2_{j}}}}\right)}^{2}\\ +\lambda_{1}||g|{|}_{1}+\lambda_{2}||d|{|}_{1}+\lambda_{1}||g|{|}_{2}+\lambda_{2}||d|{|}_{2}\\ \text{s.t. }g^{T}g=1,\;d^{T}d=1. \end{array}$$ (23)

On the basis of the optimal HR-SPLS algorithm, this study fine-tuned the parameters γ_1_and γ_2_within the range [0.01, 0.05, 0.1, 0.5, 1]. Under the optimal parameters (λ_1_ = λ_2_ = β_1_ = β_2_ = 0.5), the algorithm yielded the minimum module error for module 14. The mean of module errors, mean of module correlations, and objective function value were found to be 2.1395, 0.6810, and 28.9895, respectively.

### Diagnostic model construction

**Figure 6:**
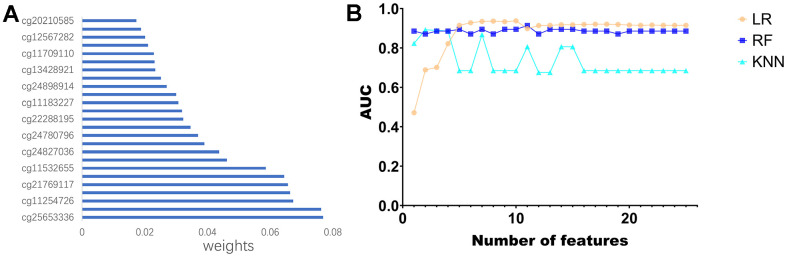
**Construction of the diagnostic model.** (**A**) Feature ranking of methylated sites using random forest algorithm. (**B**) Line graph of ranked features using three classifiers.

First, KM analysis was performed on the 105 methylation sites in module 2 to screen out the methylation sites associated with OS in PCa patients. Next, based on the random forest (RF) algorithm, the feature weights were assigned to 25 methylation sites (Supplementary Figure 2) associated with the prognosis of PCa patients (Figure 6A). Further, this paper uses the logistic regression (LR) algorithm, the RF algorithm, and the K-Nearest Neighbor (KNN) algorithm to construct the diagnosis model of prostate cancer. Specifically, we used different numbers of Top features, put them into three classifiers, and compared the AUC of the classifiers (Figure 6B).

Use the characteristics of 10 top LR algorithm (cg20210585 cg12567282, cg11709110, cg13428921, cg24898914, cg11183227, cg22288195, cg24780796, cg24827036, cg11532 655, cg21769117 cg11254726, classify cg25653336), on the test set can reach the highest AUC for the 0.9378. To further validate the effectiveness of the algorithm, this paper introduces two non-negative matrix factorization (NMF) based algorithms, namely MDJNMF [12] and JDSNMF [14]. The ROC curves of the diagnostic models constructed by these two algorithms are presented in Supplementary Figure 1. The diagnostic model constructed by our algorithm achieved the highest AUC.

### Construction of mRNAs-related prognostic model

We extracted expression data for 104 mRNAs in Module 2 and clinical information from PCa patients. First, a univariate Cox regression analysis was performed on the expression data of mRNAs in the TCGA-PRAD cohort. According to the p-value of less than 0.03, we screened and obtained 31 mRNAs related to DFA in PCa patients (Supplementary Table 1 and Supplementary Figure 3). Next, we performed a multivariate Cox regression analysis on 31 mRNAs to construct a prognostic model. Finally, we obtained a prognostic model (Supplementary Table 2) associated with 7-mRNAs (MYH11, ACTG2, DDR2, CDC42EP3, MARCKSL1, LMOD1 and MYLK), the risk score of the prognostic model is equal to expression level of MYH11* (-3.648) + expression level of ACTG2* (-4.820) + expression level of DDR2* (-2.740) + expression level of CDC42EP3* (-3.481) + expression level of MARCKSL1 *(0.845) + expression level of LMOD1* (7.614) + expression level of MYLK* (3.542).

**Figure 7:**
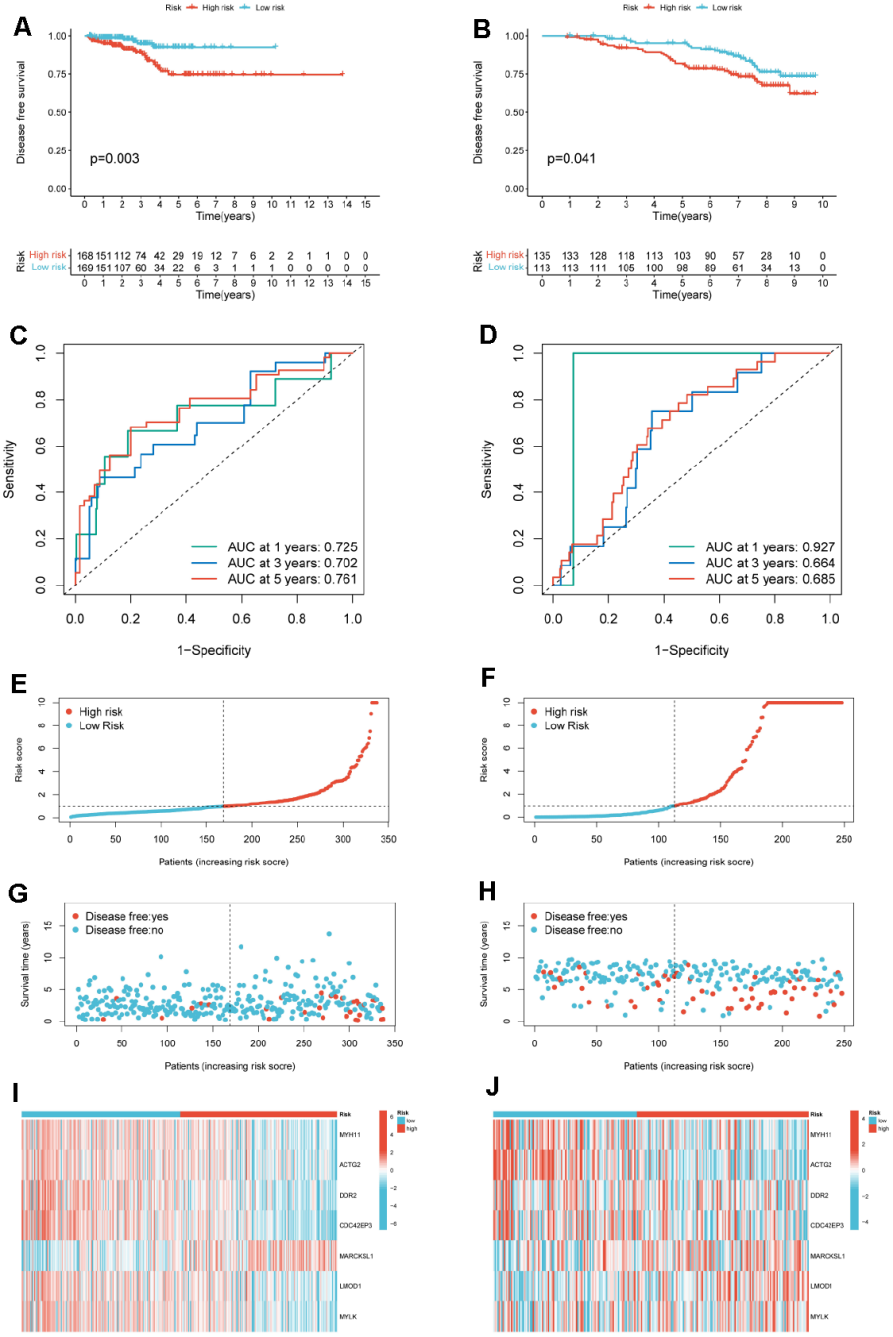
**Construction of a risk model for PCa patients.** (**A**) KM curves for PCa cancer patients in the high-/low-risk group in TCGA-PRAD. (**B**) KM curves for PCa cancer patients in the high-/low-risk group in GSE116918. (**C**) ROC curves of the risk model of 1-, 3-, and 5-years for DFS for the TCGA-PRAD. (**D**) ROC curves of the risk model of 1-, 3-, and 5-years for DFS for the GSE116918. Distribution of the risk score for TCGA-PRAD (**E**) and GSE116918 (**F**). Scatter plot of disease free status and risk score for TCGA-PRAD (**G**) and GSE116918 (**H**). Heatmap of the expression profile of the 7-mRNAs in TCGA-PRAD (**I**) and GSE116918 (**J**).

We divided PCa patients in the TCGA-PRAD cohort and GSE116918 into high and low-risk subgroups based on the median risk score. The results of the KM analysis showed that patients in the high-risk group in the TCGA cohort (p=0.003) and the GEO (p=0.041) cohort had significantly shorter DFS (Figure 7A, 7B). We further assessed the prognostic model’s predictive accuracy using the ROC curve’s AUC area. The results showed that the constructed 7-mRNAs model could predict the 1-year (AUC=0.725), 3-year (AUC=0.702), and 5-year survival rates (AUC=0.702) of PCa patients in the TCGA cohort with high accuracy 0.761) (Figure 7C). In the external dataset, the AUCs at 1, 3, and 5 years were 0.927, 0.664, and 0.685 (Figure 7D). These results demonstrate that the risk scoring model, validated in the test set, can be used to predict DFS in PCa patients. We also created heatmaps of risk factors in the TCGA cohort (Figure 7E–7G) and the GEO cohort (Figure 7H–7J). The results showed that our risk score divided PCa patients into two risk subgroups, with high-risk patients having a shorter survival time than low-risk patients. MYH11 and ACTG2 were lowly expressed in the high-risk group, while MARCKSL1 was highly expressed in the high-risk group.

**Figure 8:**
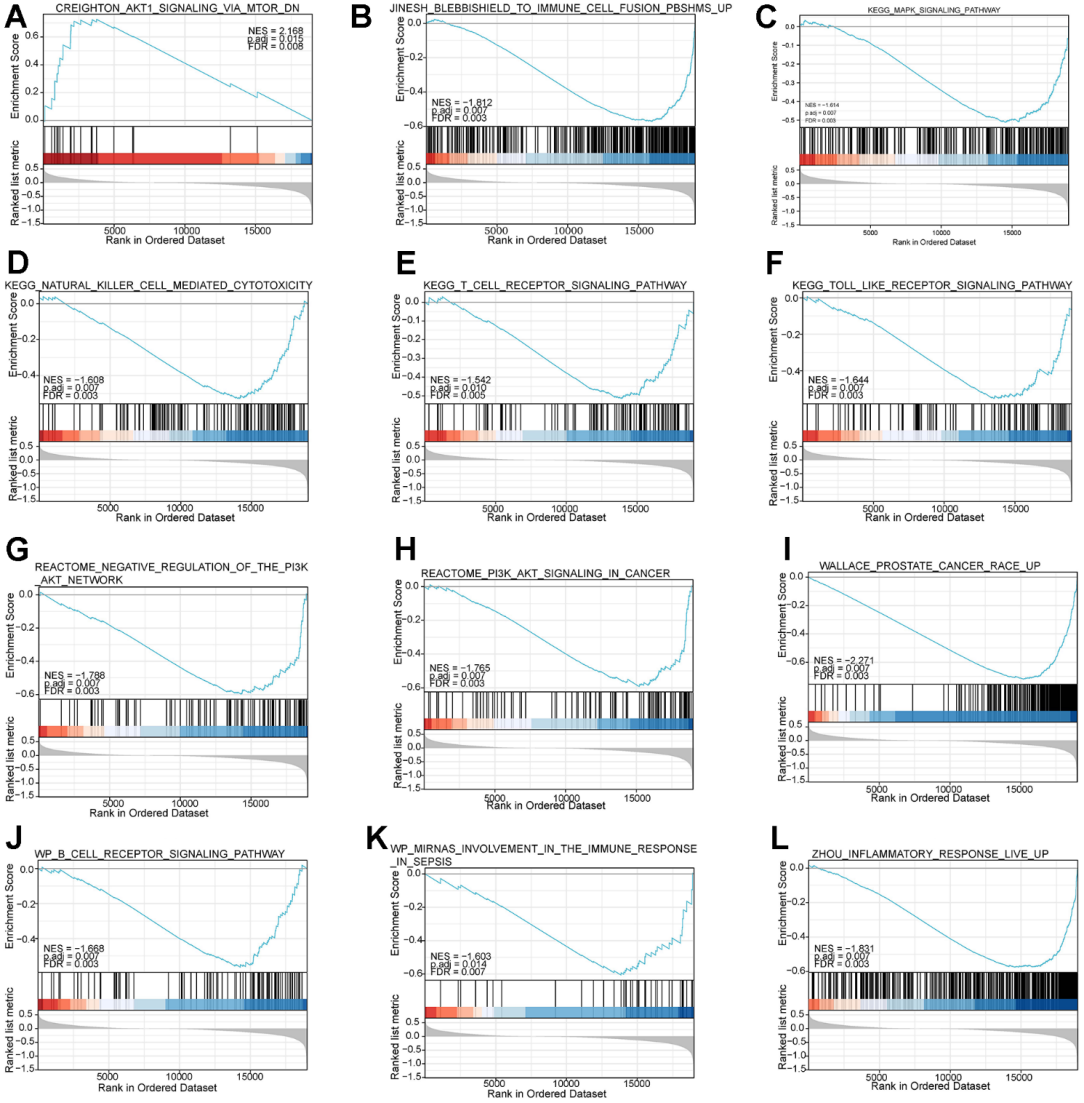
**Functional enrichment analysis based on the risk model of the 7-mRNAs by GSEA.** (**A**–**L**) give information on top pathways enriched in the high- and low-risk groups.

In addition, to explore enriched biological pathways between the two risk subgroups, we performed GSEA analysis on high and low-risk subgroups. The enrichment analysis showed that the high and low-risk groups were mainly enriched in immune and inflammation-related pathways (Figure 8A–8L), such as KEGG_NATURAL_KILLER_CELL_MEDIATED_CYTOTOXICITY, WP_B_CELL_RECEPTOR_SIGNALING_PATHWAY, KEGG_TOLL_LIKE_RECEPTOR_SIGNALING_ PATHWAY, and REACTOME_PI3K_AKT_SIGNALING_IN_CANCER.

### Analysis of the immune microenvironment among risk subgroups

We performed an immune infiltration analysis of the TCGA-PRAD cohort using the CIBERSORT method to further explore the immune microenvironment between the two risk groups. First, we calculated the proportion of 22 types of immune cells in high- and low-risk patients (Figure 9A). Next, the correlation of 22 immune cells in the TCGA cohort was explored. Dendritic cells activated and T cells CD4 memory activated had the strongest positive correlation (cor=0.42), and T cells CD4 memory resting had a strong negative correlation with T cells CD8 (cor=-0.38) (Figure 9B). Subsequently, this paper compared the infiltration levels of 22 types of immune cells in high and low-risk groups (Figure 9C). The results showed that a variety of immune cells were different between high and low-risk groups, including T cells CD4 memory resting, T cells follicular helper, T cells regulatory (Tregs), NK cells activated, Monocytes, Macrophages M0, Mast cells activated, Eosinophils, Neutrophil, and Dendritic cells resting. Among them, the infiltration level of T cells CD4 memory resting, Monocytes, Dendritic cells resting, and Mast cells activated in the high-risk group was lower in the low-risk group. In addition, this paper compared the differential expression of 47 immune checkpoints between high and low-risk groups, and a total of 33 immune checkpoints were differentially expressed (Figure 9D). Finally, the correlation between seven prognosis-related mRNAs and immune cells was explored (Figure 10A–10Y and Supplementary Figure 4). ACTG2 was positively correlated with Mast cells resting (R=0.16, p=0.0026) but negatively correlated with Macrophages M1 (R=−0.24, p=7.7e−06). CDC42EP3 was positively correlated with T cells CD4 memory resting (R=0.23, p=1.6e−05). DDR2 was positively correlated with B cells naive (R=0.21, p=0.00012). LMOD1 was negatively correlated with Macrophages M1 (R =−0.22, p = 4.4e−05). MARCKSL1 was positively correlated with Macrophages M0 (R=0.19, p=0.00038). MYH11 was negatively correlated with Macrophages M1 (R=−0.17, p=0.0021). MYLK was positively correlated with T cells CD4 memory resting (R=0.23, p=2.4e−05). These results suggest that these immune cells play a crucial role in tumor progression. In addition, we calculated the correlations of T-stage, N-stage, and Gleason score with relation to immune features (“gleason_score.cor_immune “folder in the Supplementary Material). The outcomes revealed a notable correlation between the Gleason score and Macrophages M2, Plasma cells, and T cell regulation (Tregs). Subsequently, we employed the Wilcoxon method to determine differences in distinct immune cells (retaining those with higher immune abundance) across different T and N stages. The findings indicated significant differences in T cell regulation (Tregs) during the N-stages and notable variances in Plasma cells during the T-stages. These results suggest a pivotal role for T cell regulation (Tregs) and Plasma cells in the immune microenvironment of Pca.

**Figure 9:**
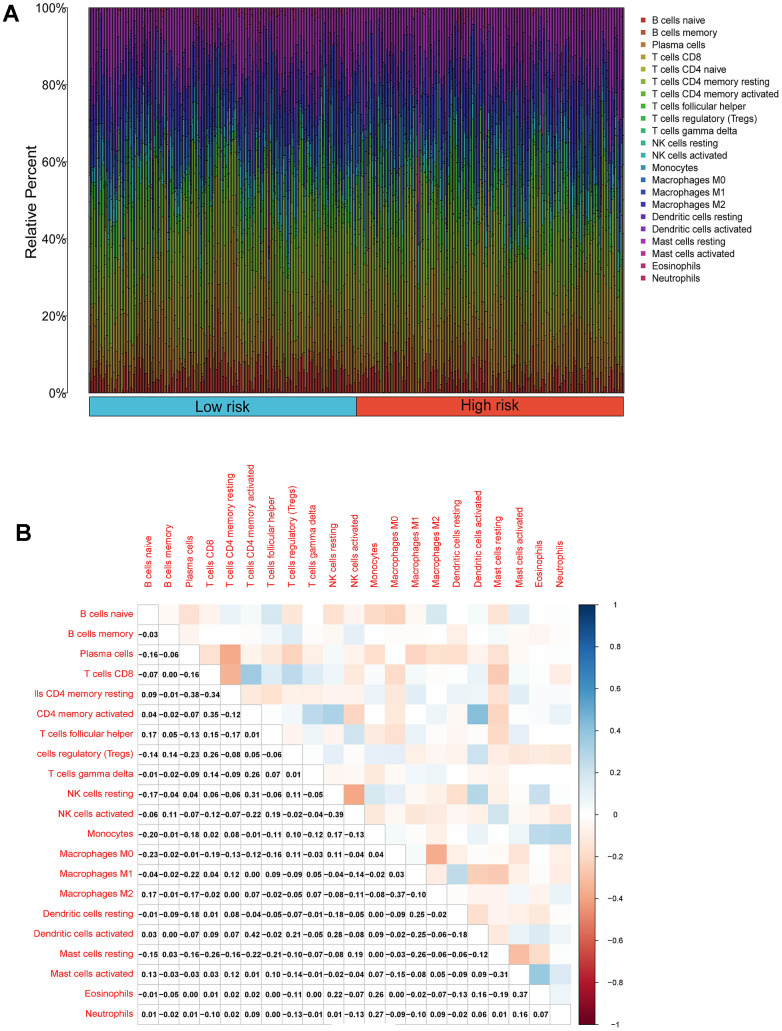
**Compositions of infiltrated immune cells between low-risk and high-risk groups in TCGA-PRAD.** (**A**) Abundance of 22 immune cell types in TCGA-PRAD. (**B**) Correlation heatmap of the immune cells.

**Figure 9:**
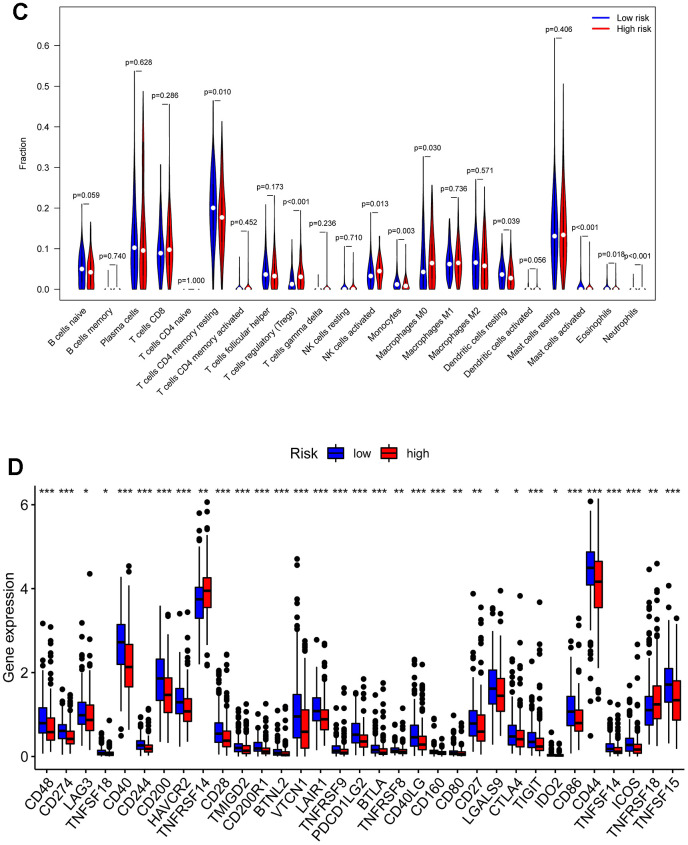
**Compositions of infiltrated immune cells between low-risk and high-risk groups in TCGA-PRAD.** (**C**) Comparisons between immune cells in low-risk and high-risk groups in TCGA-PRAD. (**D**) Expression differences of immune checkpoints between high- and low-risk groups. The blue violin reflects the low-risk group and the red violin represents the high-risk group.

**Figure 10:**
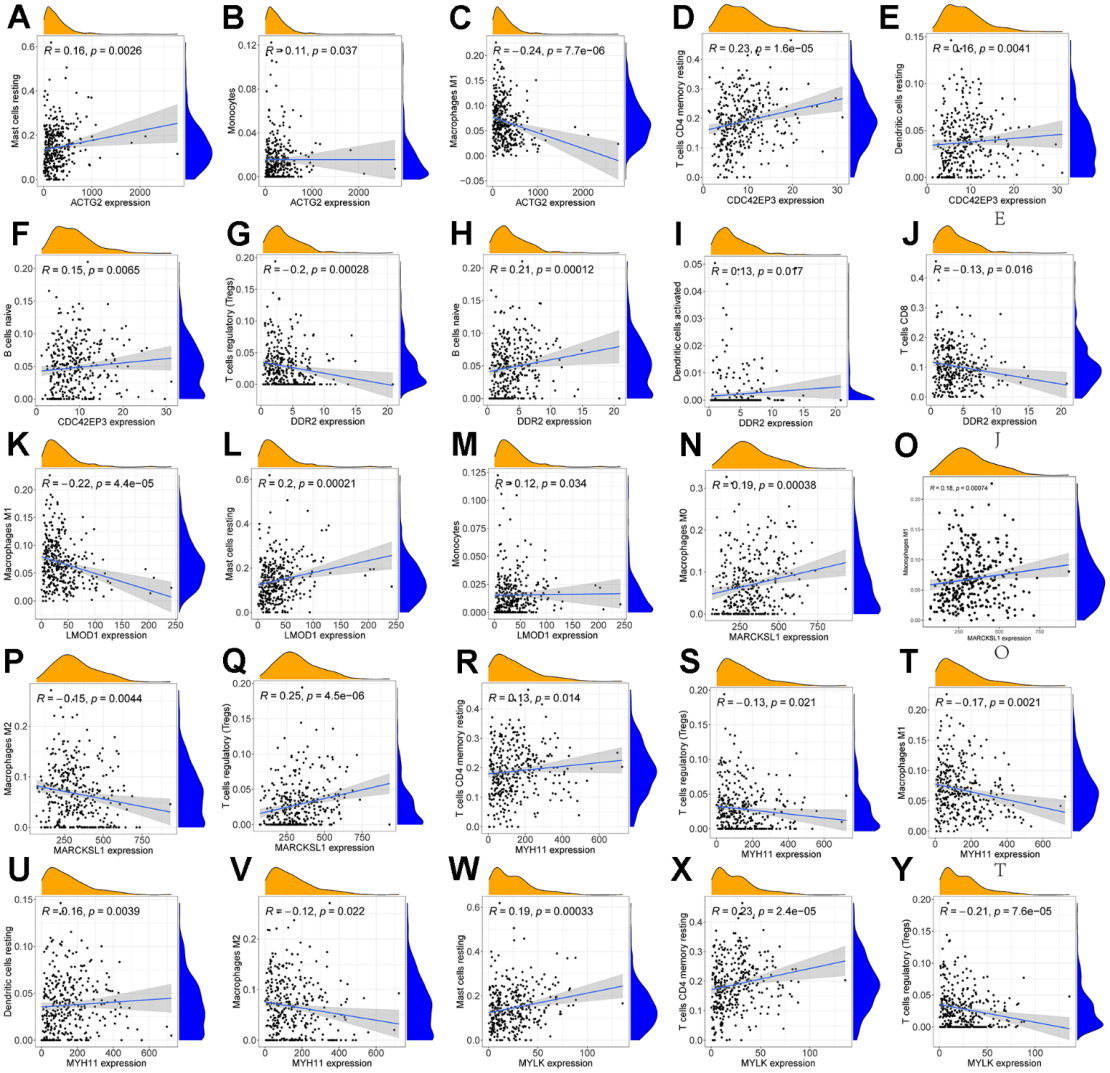
**Correlation of immune cells with 7 mRNAs associated with prognosis.** Scatter plots of the correlation between ACTG2 and immune cells are presented in (**A**–**C**). (**D**–**F**) present a scatter plot of the correlation between CDC42EP3 and immune cells. (**G**–**J**) present a scatter plot of the correlation between DDR2 and immune cells. Scatter plots of the correlation between LMOD1 and immune cells are presented in (**K**–**M**). (**N**–**Q**) give a scatter plot of the correlation between MARCKS1 and immune cells. (**R**–**U**) produced a scatter plot of the correlation between MYH11 and immune cells. (**V**–**Y**) present a scatter plot of the correlation between MYLK and immune cells.

## DISCUSSION

DNA methylation plays an essential role in regulating gene expression. It is actively involved in the occurrence and development of diseases [15]. Therefore, this paper aims to screen important PCa-related biomarkers by integrating DNA methylation and gene expression data.

First, this paper proposed the HR-SPLS algorithm to integrate the two kinds of data. Compared with the original SPLS algorithm and SNPLS algorithm, the module correlation of our proposed HR-SPLS algorithm is better than the other two algorithms. The mean value of the module error is between the two different algorithms, which further confirms the correlation performance of the algorithm on the two kinds of data. According to the corresponding weights of each module, the corresponding *u* and *v* of module 2 have higher weights and more minor errors. Therefore, we selected 105 DNA methylation sites and 104 mRNAs in module 2 for further analysis.

Then, KM analysis was performed on 104 DNA methylation sites, and 25 DNA methylation sites related to OS of PCa patients were obtained. To further screen the key DNA methylation sites in PCa, the LR, RF, and KNN algorithms were used to construct a DNA methylation site-specific PCa diagnostic model. The results showed that the top 10 methylation sites (CG20210585, CG12567282, CG11709110, CG13428921, CG24898914, CG11183227, cg22288195, cg24780796, Cg24827036, CG11532655, CG21769117, CG11254726, CG25653336) could achieve the maximum AUC of 0.9378 on the test set. Subsequently, we performed prognostic survival analysis on 105 mRNAs and constructed a prognostic model related to 7-mRNAs (MYH11, ACTG2, DDR2, CDC42EP3, MARCKSL1, LMOD1, and MYLK). The results of the ROC analysis showed that the prognostic model had high prediction accuracy (AUC=0.761). In addition, the external data set also verified the prediction accuracy of the prognostic model (AUC=0.685). GSEA analysis showed that the pathways enriched between the high and low-risk groups were mainly related to immunity and inflammation. Therefore, this paper further explored the immune microenvironment of patients in the two risk groups. We found that the infiltration levels of various immune cells differed between the high and low-risk groups, such as T cells CD4 memory resting, Tregs, NK cells activated, and Macrophages M0. CD4+ T cells can reduce the drug sensitivity of PCa patients by regulating CCL5 signaling [16]. *In vivo* and *in vitro* experiments have found that Tregs can inhibit anti-tumor responses and increase the risk of cancer recurrence [17]. Christine Pasero et al. found that NK cells from PCa patients with long postoperative survival time showed high activated receptor expression and cytotoxicity, suggesting that NK cells may become predictive biomarkers for PCa patients [18]. The above results indicate these immune cells may be essential in developing PCa patients.

MYH11 is a crucial regulator of smooth muscle contraction. MYH11 contained a frameshift mutation c.5798delC in PCa patients, possibly leading to a protein with unregulated motor activity [19]. Chen et al. showed that MYH11 and ACTG2 are potential biomarkers affecting DFS in PCa patients [20]. This is consistent with our results, and we also found that MYH11 expression was lower in the high-risk group. Abnormal expression of ACTG2 has been found in many cancers, such as ACTG2 involved in cell migration and distant metastasis in liver cancer [21]. Our results found that the expression of ACTG2 was lower in the high-risk group of patients. Azemikhah et al. found that the expression level of DDR2 in PCa tissues was significantly higher than in adjacent normal tissues and was significantly correlated with the clinical stage [22]. In PCa cells, on the one hand, the low expression of DDR2 promotes the proliferation of osteocytes. On the other hand, the overexpression of DDR2 accelerates the differentiation of osteocytes [23]. The above results suggest that DDR2 is associated with tumor metastasis in PCa cancer patients. Previous experiments showed MicroRNA-141 could hinder tumor growth and metastasis in PCa by regulating CDC42EP3 [24]. MARCKSL1 is one of the targets of miR-21. miR-21 is significantly associated with tumor growth and metastasis in various cancers [25]. In PCa, MARCKSL1 is strongly induced and up-regulated, and the knockdown of MARCKSL1 affects actin stability and migration in cancer cells [26]. Luo et al. identified LMOD1 as a biomarker associated with PCa prognosis [27]. Rebeca Kawahara et al. identified LMOD1 as a candidate biomarker of PCa aggressiveness based on the Gleason score of PCa tissue biopsies [28]. MYLK can promote PCa progression by regulating the expression of miR-29a [29]. Peng Qiao et al. used a machine learning approach to identify MYLK as a robust biomarker associated with postoperative PCa recurrence [30]. The above results indicate that the 7-mRNAs obtained in this paper are critical genes related to PCa metastasis and may provide new targets for treating PCa patients. The GSE116918 dataset utilized in this study comprises transcriptomic and clinical data of prostate cancer patients, including Gleason scores and T stages. In Supplementary Figure 5, we present expression heatmaps of prognostic gene signatures selected by our algorithm across different Gleason scores and T stages. As depicted in the figures, with increasing Gleason scores, MYH11 and ACTG2 exhibit a downregulation trend, while MARCKSL1 shows an upregulation trend. The expression patterns of these three genes may be associated with lethal prostate cancer.

HR-SPLS is an effective algorithm for integrating multi-omics data, demonstrating superior biomarker identification performance for datasets with small sample sizes and high feature dimensions. To further illustrate the effectiveness of our algorithm in scenarios with large sample sizes, we examined the algorithm’s computational time under conditions where the number of features remained constant while the sample size increased. Specifically, we randomly generated two types of omics data matrices, maintaining other parameters constant, with sample sizes set at 500, 1000, 2000, and 5000, respectively. The algorithm’s computational times were 5 seconds, 20 seconds, 46 seconds, and 113 seconds, corresponding to the aforementioned sample sizes. This further confirms the algorithm’s scalability in situations with larger sample sizes.

## CONCLUSIONS

PCa is a malignant tumor, and its early diagnosis is necessary. This paper proposes an HR-SNPLS model to integrate gene expression data and methylation data of prostate cancer, and the maximum AUC of the constructed diagnostic model is 0.9378. In addition, this paper performed prognostic survival analysis of mRNAs in the signature module. We constructed a prognostic model of 7-mRNAs associated with PCa DFS. ROC analysis validated the predictive accuracy of the prognostic model in the TCGA and GEO cohorts. In future research, we will try to integrate more types of data and expand the algorithm’s usage scenarios to identify prostate cancer biomarkers more comprehensively and systematically.

## Supplementary Material

Supplementary Figures

Supplementary Tables
