# Supplementary material for: Prognosis and diagnosis of prostate cancer based on hypergraph regularization sparse least partial squares regression algorithm
Source: Aging (Albany NY). 2024 May 31;16(11):9599–624. doi: 10.18632/aging.205889 (PMC11210239; doi:10.18632/aging.205889)
Supplement: Supplementary Figures [file aging-16-205889-s001.pdf]

SUPPLEMENTARY FIGURES

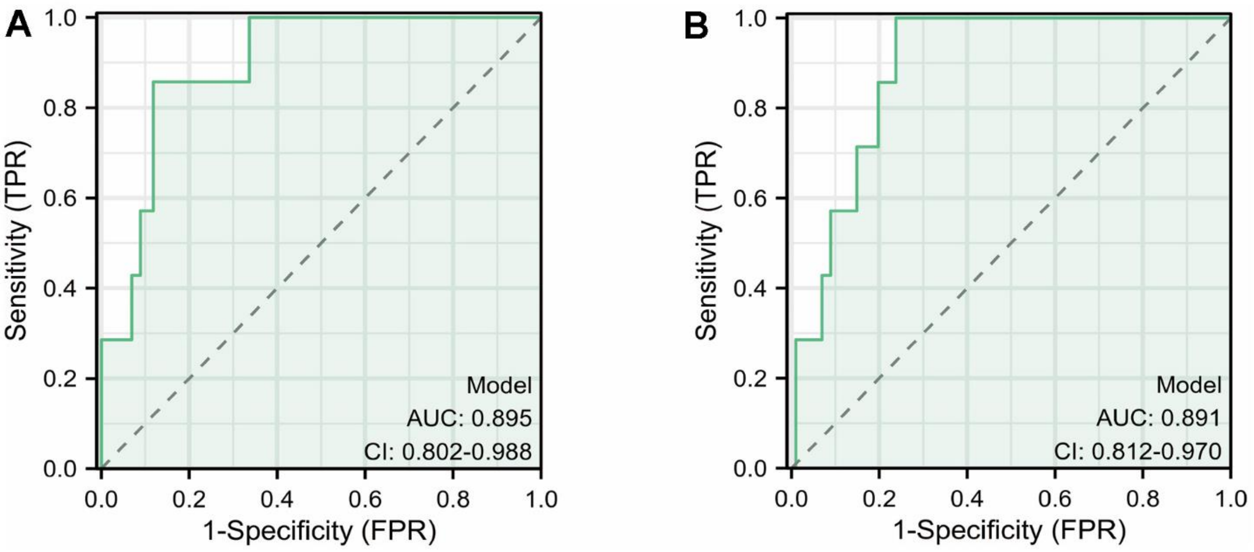

Supplementary Figure 1. The diagnostic model was constructed by the methylation markers selected by the MDJNMF algorithm and JDSNMF algorithm. (**A**, **B**) are the ROC curves of the diagnostic model constructed by the MDJNMF algorithm and JDSNMF algorithm, respectively.

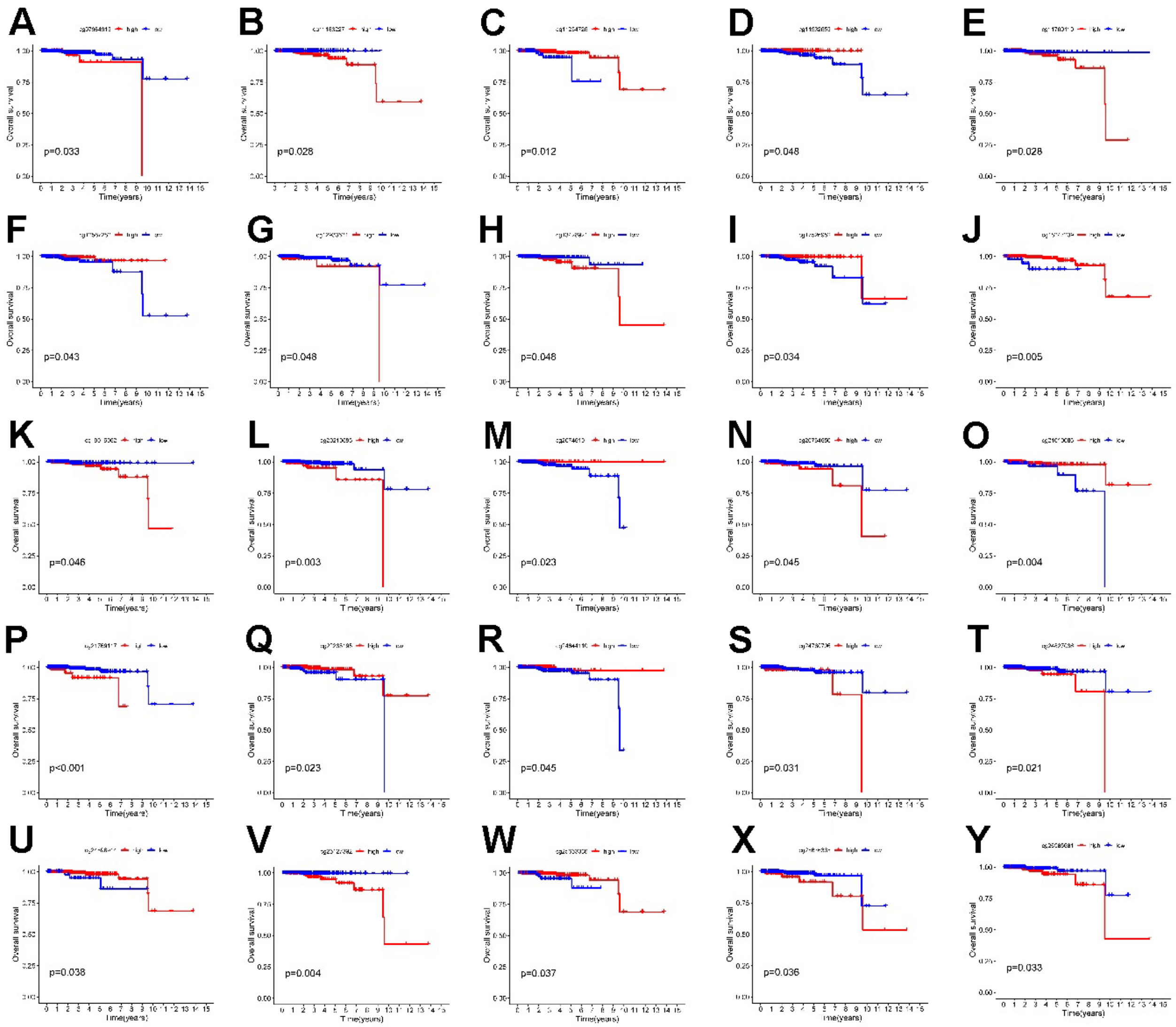

Supplementary Figure 2. Kaplan–Meier analysis of the effects of 25 DNA methylation sites on overall survival in TCGA-PRAD patients. (A–Y) are the KM survival curve of 25 methylation sites, respectively.

	pvalue	Hazard ratio
SYNPO2	0.008	0.456(0.256−0.813)
DES	0.026	0.396(0.175−0.895)
ZNF185	0.026	0.345(0.135−0.881)
RAB9B	0.011	0.373(0.175−0.795)
KCNMB1	0.022	0.389(0.173−0.872)
FLNA	0.039	0.466(0.226−0.962)
ATP2B4	0.013	0.313(0.126−0.782)
MYH11	0.010	0.389(0.190−0.796)
TLR3	0.045	0.379(0.147−0.978)
ACTG2	0.011	0.348(0.155−0.782)
DDR2	0.015	0.353(0.153−0.816)
FAXDC2	0.041	0.416(0.179−0.963)
TGFBR3	0.042	0.378(0.148−0.966)
TNS1	0.028	0.390(0.168−0.901)
CNN1	0.031	0.506(0.273−0.938)
CDC42EP3	<0.001	0.125(0.043−0.368)
MEIS1	0.036	0.397(0.167−0.943)
PRICKLE2	0.019	0.318(0.122−0.831)
HSPB8	0.010	0.451(0.247−0.824)
AOC3	0.022	0.422(0.201−0.885)
ANO6	0.033	0.382(0.158−0.923)
GPR161	0.026	0.345(0.135−0.882)
MARCKSL1	0.038	3.151(1.068−9.294)
LMOD1	0.042	0.461(0.219−0.973)
SLC24A3	0.044	0.355(0.129−0.974)
MYLK	0.022	0.415(0.196−0.879)
CAV2	0.049	0.410(0.169−0.995)
C3orf70	0.010	0.377(0.180−0.789)
PTGIS	0.008	0.401(0.205−0.786)
PDGFC	0.011	0.386(0.184−0.807)
PLN	0.022	0.508(0.285−0.905)

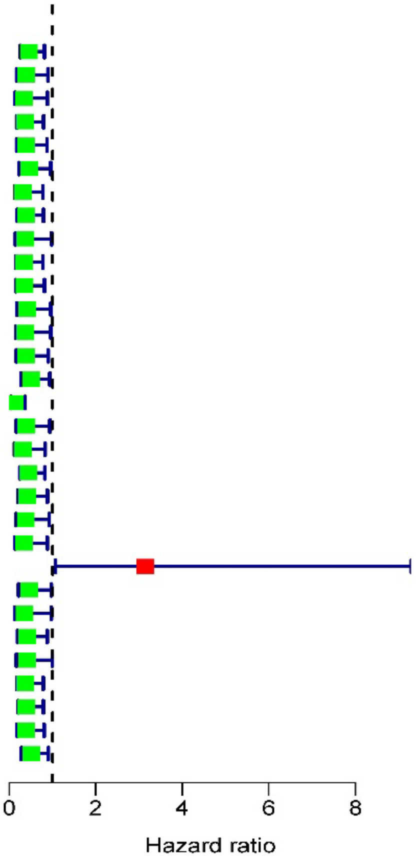

Supplementary Figure 3. The univariate Cox regression analysis results show the 31 prognosis-related mRNAs.

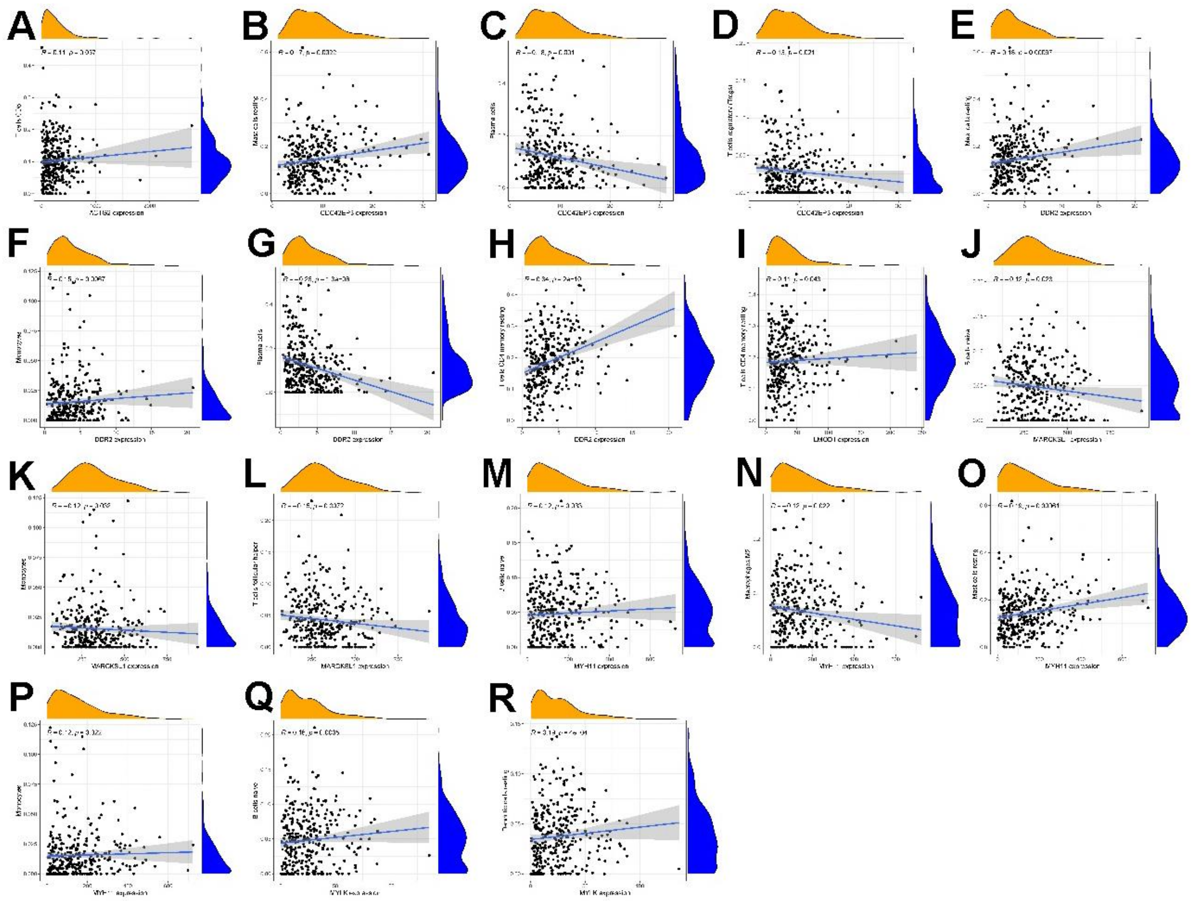

Supplementary Figure 4. Correlation of immune cells with 7 mRNAs associated with prognosis. **(A–R)** are scatter plot of 7 mRNAs and immune cell correlation, respectively.

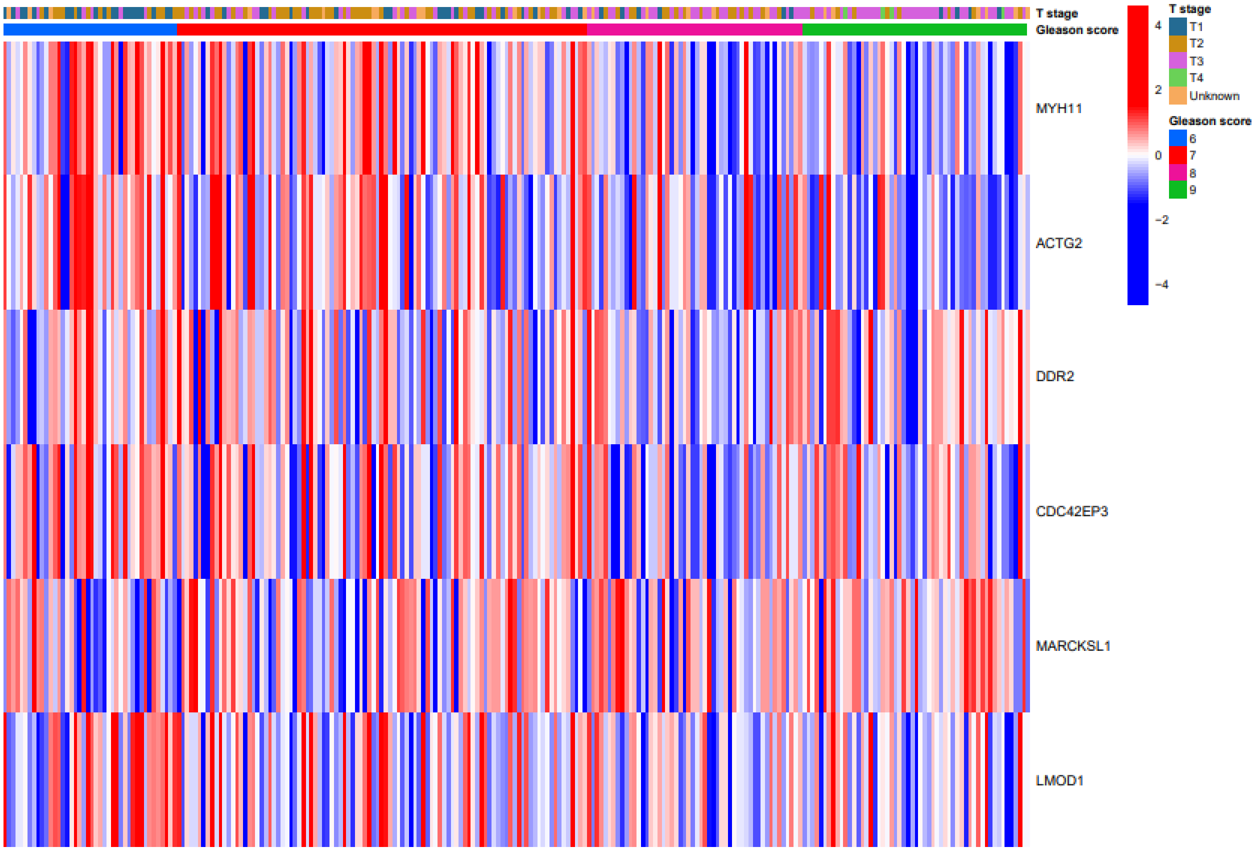

Supplementary Figure 5. Heat map of prognostic gene expression versus T stage and Gleason score in prostate cancer samples from the GEO cohort.
