# Supplementary material for: Prognosis and diagnosis of prostate cancer based on hypergraph regularization sparse least partial squares regression algorithm
Source: Aging (Albany NY). 2024 May 31;16(11):9599–624. doi: 10.18632/aging.205889 (PMC11210239; doi:10.18632/aging.205889)
Supplement: Supplementary Tables [file aging-16-205889-s002.pdf]

SUPPLEMENTARY TABLES

Supplementary Table 1. 31 mRNAs associated with PCa DFS time were obtained from univariate Cox regression analysis.

Id	HR	HR.95L	HR.95H	p-value
SYNPO2	0.456414	0.256276	0.812851	0.00773
DES	0.395982	0.175261	0.894675	0.025908
ZNF185	0.345439	0.135412	0.881221	0.026108
RAB9B	0.373122	0.175214	0.794572	0.010582
KCNMB1	0.388679	0.173172	0.872373	0.021965
FLNA	0.465857	0.22569	0.961598	0.038841
ATP2B4	0.313308	0.125562	0.781776	0.012859
MYH11	0.389088	0.190243	0.795769	0.009717
TLR3	0.379091	0.146876	0.978443	0.044962
ACTG2	0.348428	0.155309	0.781682	0.010545
DDR2	0.353383	0.153033	0.816031	0.014847
FAXDC2	0.415565	0.179273	0.963303	0.040646
TGFBR3	0.378154	0.14801	0.966157	0.042164
TNS1	0.389638	0.16841	0.901481	0.027644
CNN1	0.50642	0.273275	0.938473	0.03064
CDC42EP3	0.125272	0.042677	0.367722	0.000156
MEIS1	0.397359	0.167454	0.942909	0.036323
PRICKLE2	0.31801	0.121757	0.83059	0.01934
HSPB8	0.451373	0.247129	0.824418	0.009649
AOC3	0.421767	0.201075	0.884681	0.022363
ANO6	0.381724	0.157805	0.923377	0.032611
GPR161	0.345385	0.135231	0.882127	0.026275
MARCKSL1	3.150843	1.068211	9.293867	0.037569
LMOD1	0.461383	0.218824	0.972811	0.042114
SLC24A3	0.354605	0.129147	0.973657	0.044245
MYLK	0.415042	0.195871	0.879453	0.021719
CAV2	0.409811	0.168732	0.995336	0.048805
C3orf70	0.377247	0.180398	0.788897	0.0096
PTGIS	0.401191	0.204874	0.785624	0.00773
PDGFC	0.385502	0.184146	0.807034	0.011448
PLN	0.507888	0.284961	0.905213	0.021579

HR, hazard ratio; HR.95L, low 95% CI of HR; HR.95H, high 95% CI of HR.

Supplementary Table 2. 7 mRNAs were obtained from multivariate Cox regression analysis.

Id	coef	HR	HR.95L	HR.95H	p-value
MYH11	-3.64818	0.026039	0.000337	2.012673	0.100044
ACTG2	-4.82026	0.008065	0.000179	0.363691	0.013122
DDR2	-2.74029	0.064552	0.007127	0.584631	0.014793
CDC42EP3	-3.48122	0.03077	0.00387	0.244631	0.000998
MARCKSL1	0.845069	2.328138	0.703578	7.703804	0.166321
LMOD1	7.614462	2027.304	38.06145	107982.3	0.000174
MYLK	3.542567	34.5555	2.447259	487.9266	0.008729

Coef, the coefficient of genes (MYH11, ACTG2, DDR2, CDC42EP3, MARCKSL1, LMOD1 and MYLK) correlated with DFS; HR, hazard ratio; HR.95L, low 95% CI of HR; HR.95H, high 95% CI of HR.
